# Age structure of the Australian lungfish (*Neoceratodus forsteri*)

**DOI:** 10.1371/journal.pone.0210168

**Published:** 2019-01-23

**Authors:** Stewart J. Fallon, Andrew J. McDougall, Tom Espinoza, David T. Roberts, Steven Brooks, Peter K. Kind, Mark J. Kennard, Nick Bond, Sharon M. Marshall, Dan Schmidt, Jane Hughes

**Affiliations:** 1 Research School of Earth Sciences, The Australian National University, Canberra, ACT, Australia; 2 Queensland Department of Natural Resources, Mines and Energy, Bundaberg, QLD, Australia; 3 Seqwater, Ipswich, Brisbane, QLD, Australia; 4 Queensland Department of Agriculture and Fisheries, Brisbane, QLD, Australia; 5 Griffith University, Brisbane, QLD, Australia; 6 The Murray-Darling Basin Freshwater Research Centre, Latrobe University, Albury-Wodonga, Victoria, Australia; Fred Hutchinson Cancer Research Center, UNITED STATES

## Abstract

The Australian lungfish has been studied for more than a century without any knowledge of the longevity of the species. Traditional methods for ageing fish, such as analysis of otolith (ear stone) rings is complicated in that lungfish otoliths differ from teleost fish in composition. As otolith sampling is also lethal, this is not appropriate for a protected species listed under Australian legislation. Lungfish scales were removed from 500 fish from the Brisbane, Burnett and Mary rivers. A sub–sample of scales (85) were aged using bomb radiocarbon techniques and validated using scales marked previously with oxytetracycline. Lungfish ages ranged from 2.5–77 years of age. Estimated population age structures derived using an Age Length Key revealed different recruitment patterns between river systems. There were statistically significant von Bertalanffy growth model parameters estimated for each of the three rivers based on limited sample sizes. In addition, length frequency distributions between river systems were also significantly different. Further studies will be conducted to review drivers that may explain these inter-river differences.

## Introduction

The Australian lungfish (*Neoceratodus forsteri*) is perhaps one of the world’s oldest living vertebrates, thought to survive for up to 100 years [1]. This longevity means that current populations may contain a large proportion of adults that pre–date many of the present–day threatening processes occurring across their current range in southeast Queensland [2]. Adult lungfish can persist through long periods of environmental stress, but can fail to produce recruits (offspring that reach maturity and successfully breed) over much of these stressful periods [3]. This situation can contribute significantly to extinction debt, where the chronic impacts of environmental changes on population viability are masked due to the longevity of a species and lack of understanding of recruitment processes [4]. A critical piece of information required to understand the nature of the extinction risk for a long lived species is the age structure of the population [5]. However, traditional methods for ageing fish, such as analysis of otolith (ear stone) rings are inappropriate for this species. Not only is otolith sampling lethal, lungfish otoliths do not exhibit the annual banding that is characteristic of most teleost fish species [6]. This inability to age Australian lungfish is a key knowledge gap impeding understanding of current population dynamics and conservation action.

The Australian lungfish is the most primitive member of the Dipnoi family, which is composed of only five other lungfish species (one South American and four African). Fossil evidence suggests lungfish have existed since the early cretaceous though flourished in the Devonian period (416–359 Mya) [1]. Genetic studies have revealed current populations of Australian lungfish originated from the Burnett River and later established the Mary River [7]. In addition, more recent genetic evidence supports the Brisbane River population being a product of translocations from the Mary River in the late 19^th^ century [7–8].

At present, the global distribution of Australian lungfish is restricted to southeast Queensland, an area that has experienced significant urban growth in recent years. This has also led to an increase in water resource development and potential threats to aquatic ecosystems [2]. The Brisbane and Burnett river catchments contain numerous large dams and weirs that regulate streamflow primarily for urban centres and agricultural production. Water resource development has either removed or altered significant riverine Australian lungfish spawning habitat through impoundment or flow regime changes [9–10]. In contrast, the Mary River has a low level of flow regulation and more natural flow regime [11]. Impacts to lungfish populations have been documented and concern over the sustainability of a species with proposed long term recruitment failure has been highlighted, resulting in the Australian lungfish being listed as “threatened” under the Australian *Environment Protection and Biodiversity Conservation Act*, *1999* [12].

Previous attempts to age lungfish by traditional means have failed, including the use of otoliths which requires killing specimens, something unsuitable for a threatened species [7]. Attempts to use lungfish teeth for aging failed as the teeth were found to be highly porous and unsuitable [13]. Scales have been collected from lungfish in the past, including scales marked with oxytetracycline (OTC). Visual interpretation of increments on the scales revealed that this method is likely to underestimate the age of the fish, particularly older fish and could not be validated [13]. Mark–recapture was used to produce von Bertalanffy growth parameters for the species, however growth between tag and recapture periods was limited leading to potential errors in final growth functions [13]. Because other techniques to age lungfish have failed in the past, bomb radiocarbon aging techniques were trialed on scales of this species where the results showed that appropriate source material could be found in the scales to be used for dating and that the whole of the radiocarbon chronology was visible in the scales [14–15]. Atomic testing in the 1950s and 1960s caused increases in radiocarbon (^14^C) levels in the atmosphere that have then been reflected in body tissues of fish. Radiocarbon dating has been successfully been applied to numerous fish species to confirm other aging techniques, estimate ages and to confirm longevity of long–lived fishes [16]. It has been applied to other freshwater fish species [16–18], estuarine fish [19] and pelagic and demersal finfish [20–22]. In the case of marine species, coral records are often used as reference chronology [23], however in the case of freshwater species these have relied on reference chronologies from other freshwater species from the same hemisphere [16]. Radiocarbon dating has been found particularly useful in aging another long–lived freshwater fish species, the pallid sturgeon (*Scaphirhynchus albus*), which is a threatened species that has suffered excessive recruitment failure and there are only few samples of larger older fish [16].

The objective of this study was to use bomb radiocarbon techniques on a sub–sample of scales of the Australian lungfish to confirm the longevity of the species within the Brisbane, Mary and Burnett rivers. The aim was then use these age distributions from the sub–sample to extrapolate to the population within these rivers as a first step in filling identified knowledge gaps in regarding recruitment of the species.

## Materials and methods

### Location

**Fig 1:**
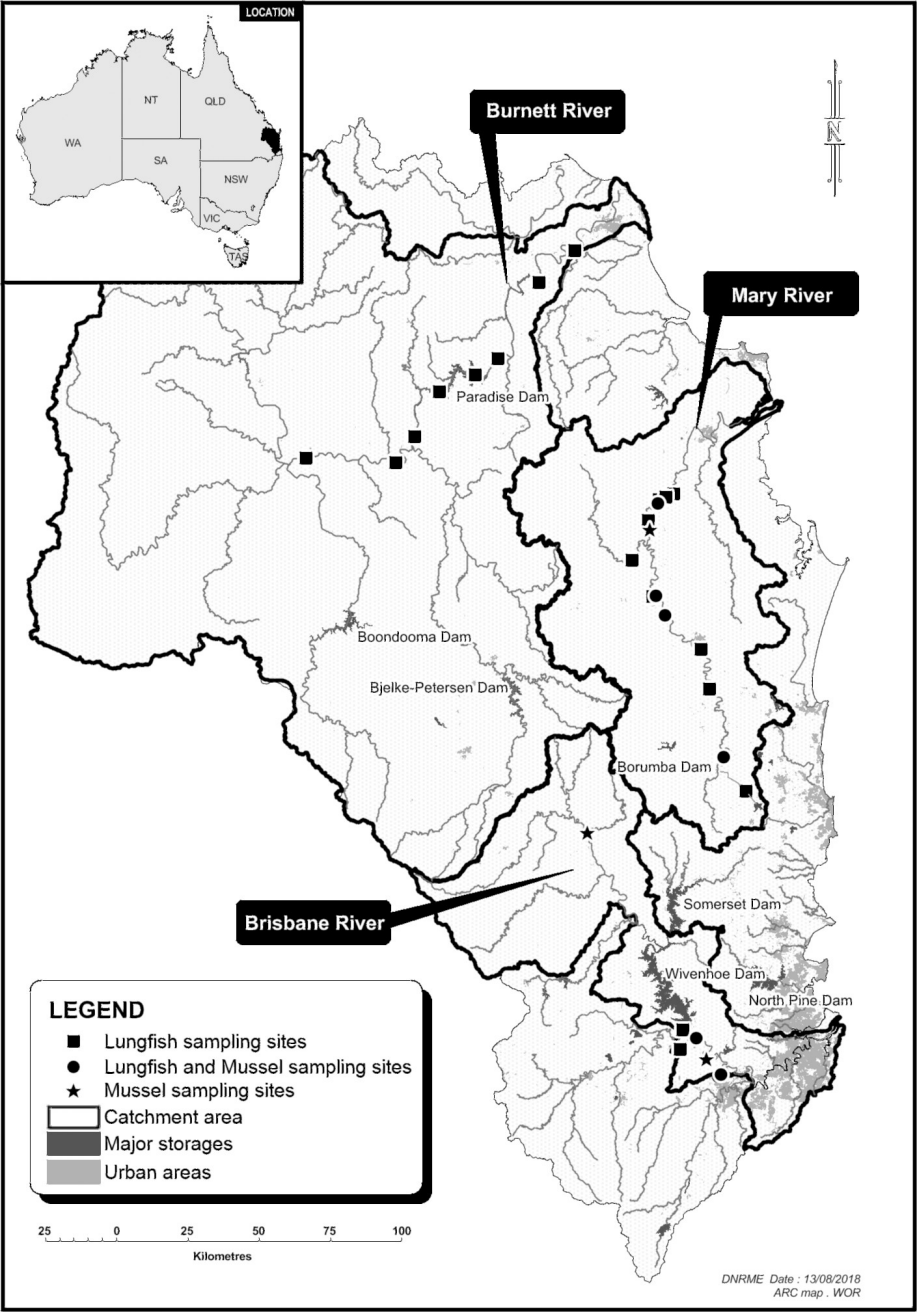
Map of fish sampling locations. The map shows the lungfish and mussel sampling locations as well as major impoundments.

Australian lungfish were collected from three rivers (Brisbane, Burnett and Mary rivers) across their current range in southeast Queensland, Australia. These three rivers are located in separate drainage divisions (Fig 1). Samples in the Burnett River were collected from riverine and impoundment sites along the length of the river. The samples in the Mary River were collected from riverine sites in the lower catchment. In the Brisbane River, samples were collected from the lower reaches of the river, downstream of Wivenhoe Dam.

### Scale collection

All field and experimental protocols carried out in this study were approved by the Griffith University Animal Ethics Committee. All procedures were carried out according to Australian Ethics Committee protocol numbers CA2011/10/551 (Seqwater) and ENV/17/14/AEC (Griffith University). Samples were collected under Fisheries Permit Numbers 140615 (Seqwater) and 174232 (Griffith University).

All fish were sampled using boat electrofishing units, as this is thought to be the most unbiased method [13]. Multiple scales were collected from the dorsal, ventral and lateral (behind pectoral fin) regions of similar number of fish from each river (Brisbane–n = 498, Mary–n = 488, Burnett–n = 506). Fish ranged in size from 495 mm to 1390 mm. A small piece of fin (1cm^2^) was also removed from the fish for genetic analysis [8]. The scales were removed from the fish with forceps and placed into sealed plastic bags labelled with a unique identification code that was matched to the genetic sample for each fish. The plastic bags were refrigerated in the field, then frozen back in the laboratory.

Due to the high cost of each ^14^C sample analysis and the need to analyze multiple samples per fish scale to determine age, a sub–sample of approximately 30 fish per river were aged. To ensure that all size ranges were sampled, two fish from each 50 mm size length class were randomly selected from each of the three rivers [24].

The sub–sampled scales were defrosted, cleaned thoroughly in water and viewed under transmitted light to discard potential regrowth scales (scales that have been lost and are regrown). Regrowth scales, when observed, lacked consistent incremental lines and contained a homogenous growth band that extended well out from the primordium towards the edge of the scale. A scale was chosen for analysis from each of the fish that showed clear growth increments through to the outer edge and there was a general consistency in growth pattern with other scales from the same fish. The primordium and the unique identifier code were marked on the outer edge of selected scales with a pencil. The pencil mark was subsequently removed during mechanical cleaning. The scales were prepared for analysis by placing the wet scales in a custom-made acrylic press (150x200 mm) ventilated with numerous drill holes and held together with bolts to ensure that the scales remained flat. The press was placed in a drying oven (Clayson OM1000ME) at 40°C for 3 days until the scales were fully desiccated. Once dried, the scales were placed in individual clear plastic bags (labelled with the unique identification number) and sent to the lab for further processing.

Additional fish were aged separately in order to (1) support genetic analyses (all rivers), (2) validate the technique using marked (oxytetracycline) and recaptured individuals and (3) age fish smaller than 850 mm total length (TL) from the Burnett River to assess recruitment patterns in relation to environmental drivers more closely, in more recent years. The additional aging for assessing recruitment variability concentrated on smaller individuals for multiple reasons: these fish required less ^14^C samples as they were born post bomb peak; there was increased accuracy of the birth date; there was no need to sub–sample the scales in the smaller size classes as there were few individuals; and the more recent time period contained more comprehensive environmental datasets, together with data collection on lungfish spawning and habitat [9, 13, 25].

### Sampling for ^14^C

**Fig 2:**
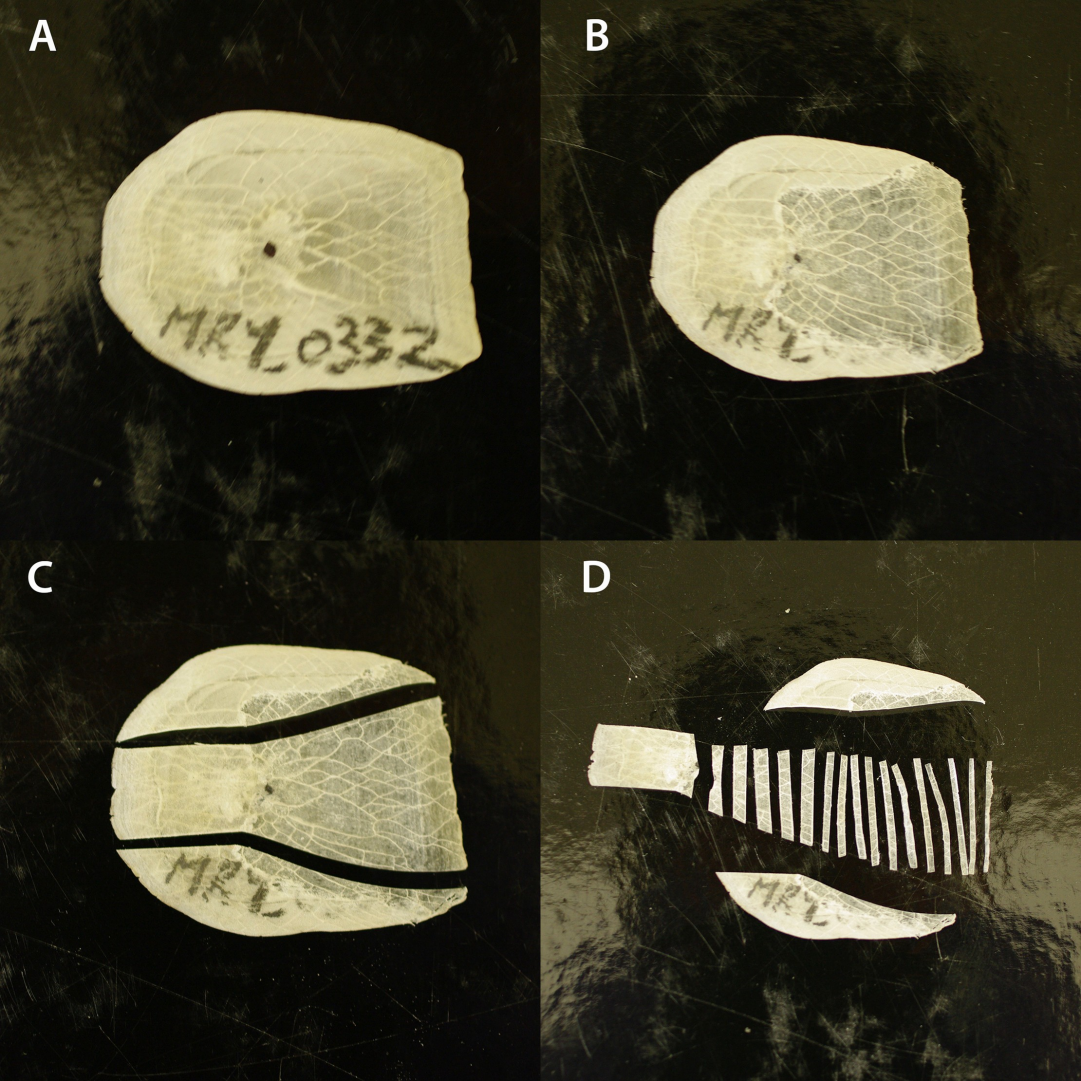
Scale sample collection. A) Dried and flat scale showing primordium as the black dot. B) Scale after removal of upper squamulae and lower elasmodin surfaces, cleaned area becomes transparent. C) Scale cut to prepare for sampling. D) F^14^C samples showing the first 11 samples removed in 1mm increments, then 2mm increments to the primordium.

**Fig 3:**
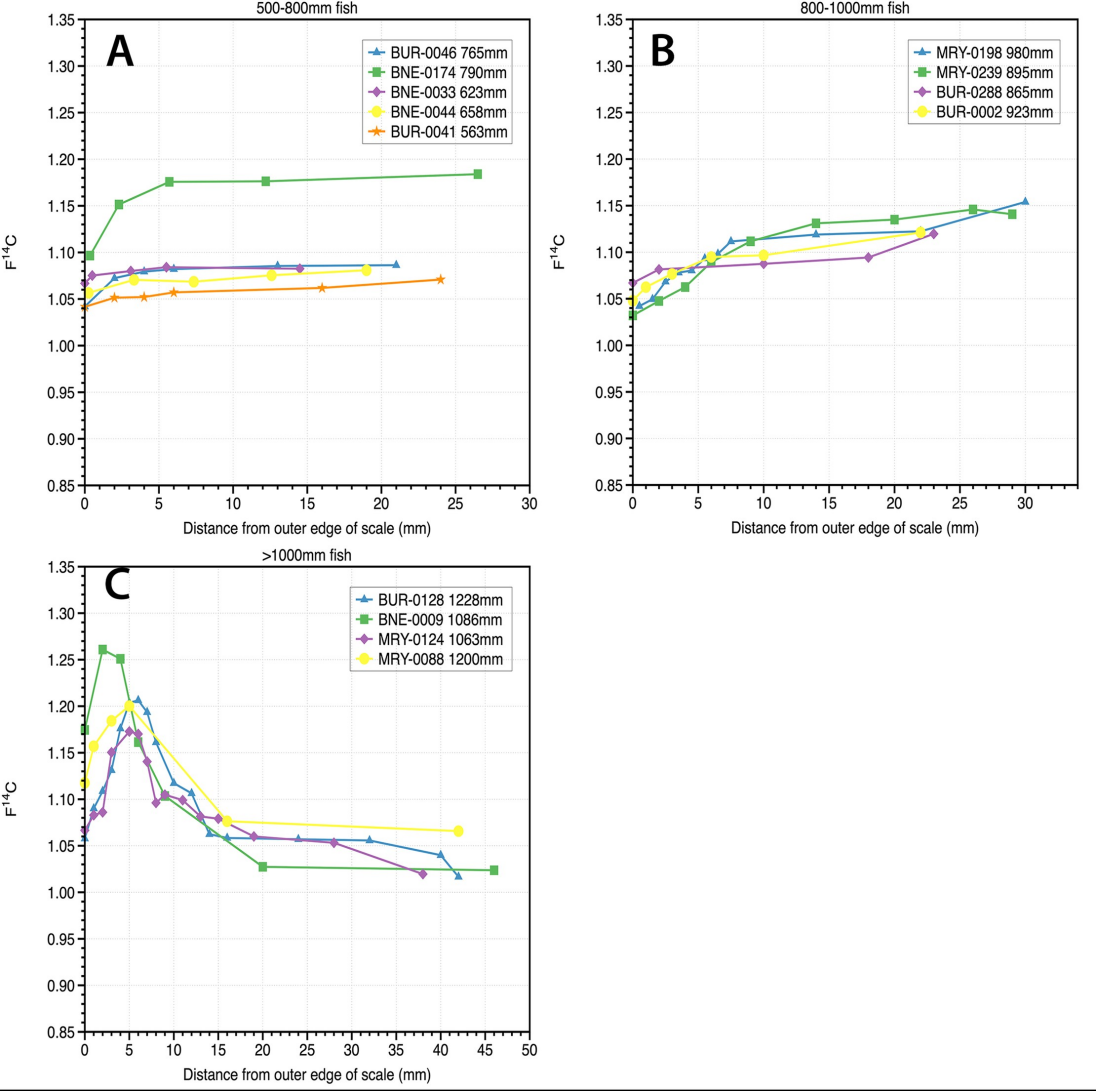
Radiocarbon results (F^14^C) from selected fish. A) Representative F^14^C from fish smaller than 800mm in length vs. distance from the outer edge of the scale B) Representative F^14^C from selected fish between 800-1000mm in length vs. distance from the outer edge of the scale. C) Representative F^14^C from fish >1000mm in length vs. distance from the outer edge of the scale. The radiocarbon bomb pulse is evident in the data from fish >1000mm in length.

Scale samples were prepared using previous methods developed for this species [14–15]. In summary, a diamond burr attached to a hand–held Dremel tool was used to remove the upper squamulae and lower elasmodin surfaces (see Figs 1 and 2 [15]). Samples were sliced into 1 mm increments near the posterior edge and 2 mm increments near the primordium (Fig 2). Approximately 1–1.5 mg of scale material was loaded into 6 mm I.D. quartz tubes with 60 mg CuO and a 4x6 mm Ag cup. The tubes were evacuated down to <10^−3^ Torr and flame–sealed. The tubes were baked at 900°C for 6 hours to produce CO_2_. Conversion to graphite was achieved in the presence of Fe powder and H_2_ gas (water being removed during reaction with Mg(ClO_4_)_2_). Samples were measured on the Single Stage Accelerator Mass Spectrometer at the Research School of Earth Sciences, The Australian National University [14]. Samples were normalized to the radiocarbon standard Oxalic Acid I and background corrected using ^14^C free coal and normalized to the accelerator mass spectrometer δ^13^C. Radiocarbon results are presented as F^14^C [26]. Approximately ten F^14^C measurements were made on each scale, with a total of ~1000 measurements made for the project. Only the F^14^C measurement from the primordium of the scale is taken forward to estimate the fish age as this material is formed near the birth of the fish. The transect of multiple slices across the scale is used to confirm whether the F^14^C value obtained at the primordium of the scale was before or after the peak of atomic testing. An example of raw radiocarbon measurements based on position on the scale are shown in Fig 3.

### Accounting for food impacts on radiocarbon dating

The age of the carbon consumed in food sources can significantly affect the F^14^C values in the body tissues of the fish being studied, particularly for deep–water fish [20]. As few freshwater fish species have been radiocarbon aged previously, several freshwater mussel species known to be prey for lungfish were sampled [27]. Mussels from the genus *Corbiculina* and *Velesunio* were collected from sites in the Brisbane and Mary Rivers between 2014–2015 to understand both between river and within–river variability of the food source F^14^C. Mussel organic samples were cleaned in 18 Mohm water, freeze dried and ~1.5 mg of sample was combusted to CO_2_ as per scales. The mussel shell carbonate was subjected to a 10% HCl acid leach, then ~8 mg of material was reacted with orthophosphoric acid under vacuum and the evolved CO_2_ was converted to graphite in the same manner as the scales.

### Validation of Method–OTC tagged fish

To validate the method of aging, a number of scales from lungfish that had been previously marked with the fluorescein dye, Oxytetracycline (OTC), were analysed. This dye assimilates into all tissues and organs that are actively metabolising [28]. In a previous study (1997–2000), approximately 1500 lungfish were injected to understand lungfish growth and longevity in the Burnett River [13]. Prior to the injection, the fish were injected with a Passive Integrated Transponder (PIT) tag as permanent identification, measured for weight, length and 3–4 scales were removed and archived [13]. During 2010–2012, 45 fish of the original 1500 injected were recaptured and re-weighed, length measured and additional scales were removed (Peter Kind, DAF pers comm., 2015). Scales from 4 fish were supplied for aging, both from the time of injection and subsequent recapture (Table 1). Approximately 10–12 F^14^C samples were measured from a scale from the originally captured fish as well as the recaptured scale.

**Table 1:** Capture/Re-capture fish data in this study.

Dart Tag No.	1^st^ Capture Date	Length (mm)	Recapture Date	Recapture Length
LF0801	30/11/98	745	17/3/10	1010
LF0901	30/11/98	810	17/3/10	1000
LF1596	21/5/99	1036	29/2/12	1060
LF1321	29/11/99	1036	26/3/09	1085

### Lungfish radiocarbon reference curve

Earlier research on scale radiocarbon ageing for Australian lungfish confirmed that the scale carbon F^14^C reproduced the shape of the atmospheric CO_2_ F^14^C, but not the absolute values [14]. The approach for bomb ^14^C dating relies on matching the shape of the scale F^14^C curve with that of a reference or atmospheric CO_2_ F^14^C curve based on known points on the curve [23]. Known time periods include the timing of the end of the pre-bomb period (1955), as well as the timing of the peak of atomic testing and atmospheric CO_2_ F^14^C (1965) [29]. The reduced amplitude of the peak F^14^C within the scale is most likely due to time–averaging of the F^14^C scale signal because of the scale sample size (widths/time) needed for the ^14^C measurement. We used a coupled–function model in order to develop a reference curve to use radiocarbon to age lungfish based on fish otolith research [21–22, 30]. The model used is shown in Eq 1.

$${\hat{y}}_{x}=\lambda +kexp\left[(u\cdot r)+\frac{\left(\sigma_{n}\cdot r\right)}{2}\right]exp(-rx)\Phi (x,a+\sigma_{n}^{2}r,\sigma_{w})+\sigma_{e}^{2}$$ Eq 1

The parameters are as follows: λ = pre–bomb ^14^C level, *k* = height of ^14^C peak, *Φ* = year of increase; *r* = slope of ^14^C decline, *σ*_n_ = slope of ^14^C increase.

**Fig 4:**
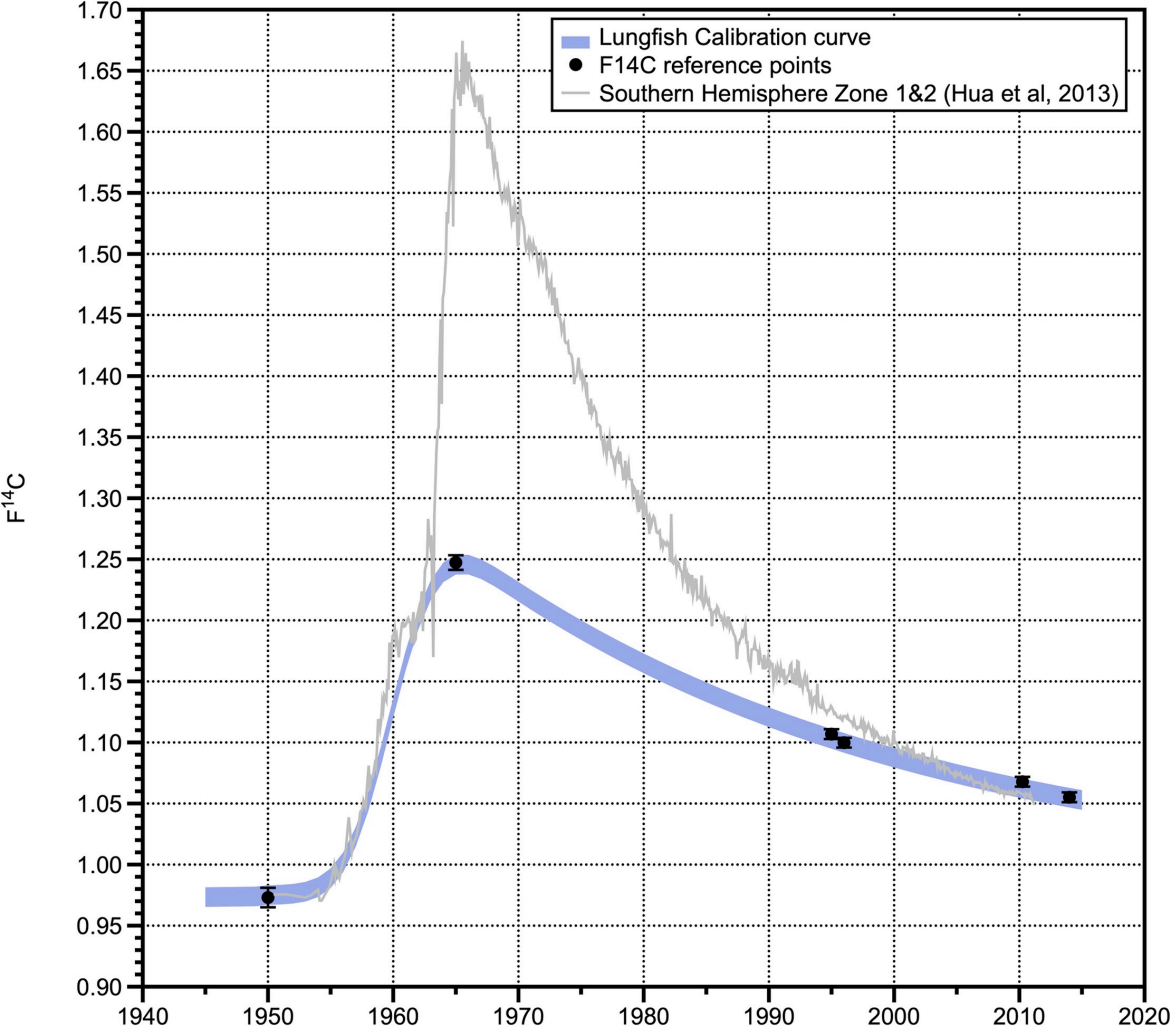
F^14^C reference curve to age Australian lungfish. Curve generated using the pre-bomb (pre-1950) ^14^C from old fish, the maximum ^14^C measured in any of the scales, and the small OTC tagged fish ^14^C from their outer slice of the scale and small fish collected in 2014.

The pre–bomb ^14^C level of 0.97 was obtained by averaging measured values from the primordium of multiple scales of fish that had stable ^14^C values predating the rise in radiocarbon curve on each scale. The peak F^14^C value was obtained from a maximum measurement of 1.24 among all scales analysed. The slope of F^14^C decline was determined from OTC mark–recaptured scales collected in 1998, 2010 and 2014. These parameters populated Eq 1 to create a reference curve for subsequent ageing of a sub-sample of the population. (Fig 4). All raw radiocarbon measurements can be viewed in S1 Table, all radiocarbon inferred ages are in S2 Table.

**Fig 5:**
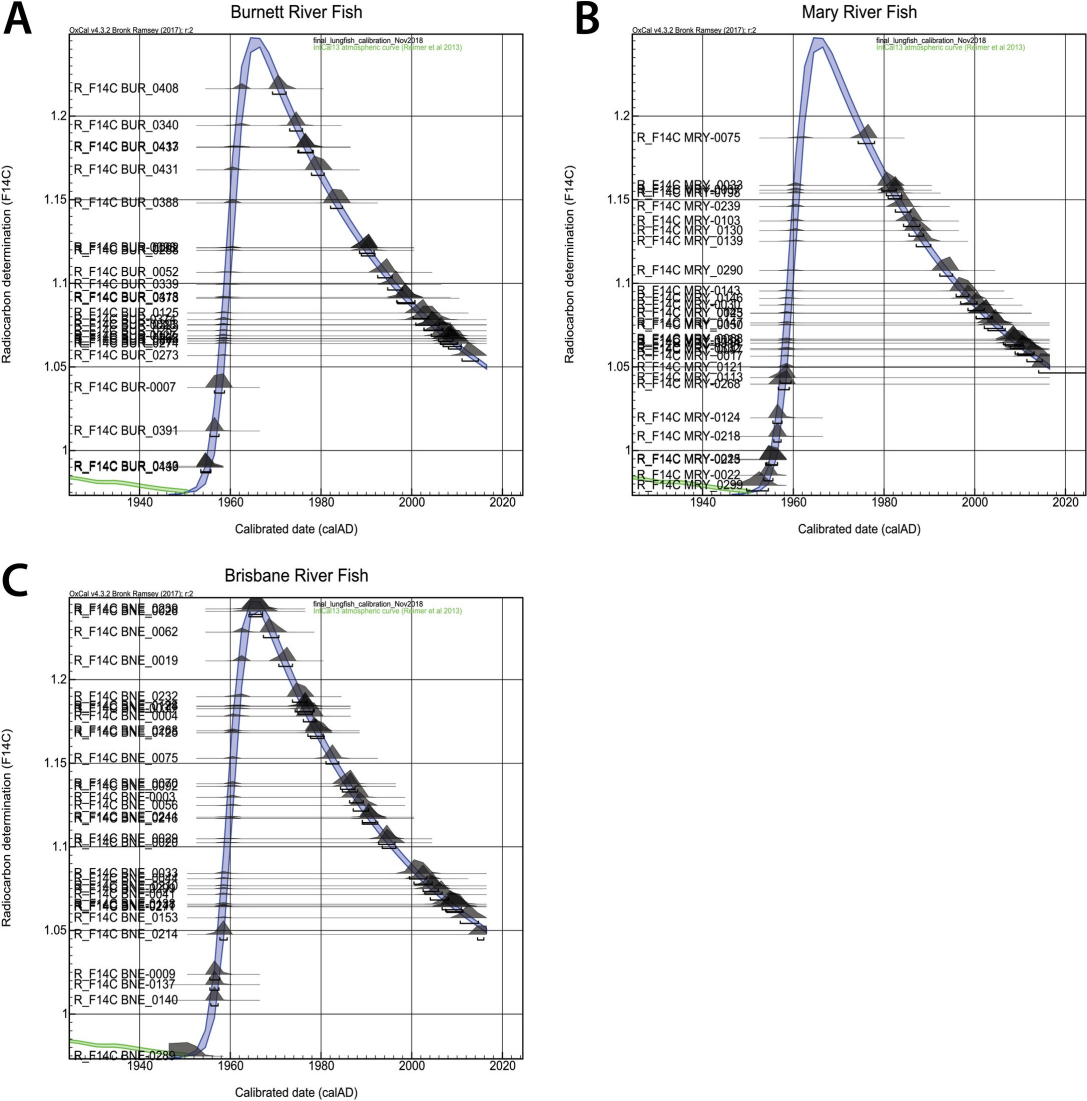
OxCal age results from all fish. A) OxCal probability distributions for lungfish ages from Burnett River overlaid on lungfish calibration curve. B) OxCal probability distributions for lungfish ages from Mary River overlaid on lungfish calibration curve. C) OxCal probability distributions for lungfish ages from Brisbane River overlaid on lungfish calibration curve.

The reference curve was then loaded into the radiocarbon calibration program (OxCal 4.2; [31]). This program is routinely used to calibrate radiocarbon measurements and provides robust age estimates and errors with the resolution of age estimates set to one to two years. The 1 sigma age ranges were then used to provide estimates of the year of birth and listed in S2 Table [14, 22, 31]. Fig 5 shows the OxCal age distributions on the lungfish calibration curve for the 30 fish from each river. For those fish with a primordium F^14^C value on the rising side of the bomb curve (1950–1963), the OxCal program was used to provide age estimates for samples at specific distances along the scale. The birth year was then calculated using a reparametrized von Bertalanffy equation using these age at length values for the individual fish [14].

To determine if the resultant age distributions for the sub–sampled populations in the rivers was different, age data were analyzed with a 2–sample Kolmogorov-Smirnov test and a Kruskal–Wallis test with pairwise Mann–Whitney tests [32].

### Comparing lungfish growth parameters and size between river systems

A standard 3 parameter von Bertalanffy growth function (VBGF) was used to describe the growth of lungfish aged by radiocarbon dating from each river system separately due to genetic differentiation [7]. The length and radiocarbon age data from each river was first pooled and the fit of the model was compared to that of the three separate river models. To determine if the pooled model adequately described the growth across rivers, the Akaike’s information criterion with an incorporated bias correction algorithm (AIC_c_) was used to compare against all river models, as this is suited for studies with low sample sizes [33]. In addition, an F–statistic was also calculated to test significance [34].

A 2 parameter VBGF curve was also produced to directly compare against previous age–length studies conducted on Australian lungfish [12]. The most parsimonious age–based VBGF for the Burnett River was determined using the same AIC_c_ statistic. Resultant VBGF models were not used in assigning ages to unaged individuals. This was done using an age-length key (ALK) described below.

The length frequency distributions between river systems were compared using 2-sample Kolmogrov-Smirnov tests [32].

### Extrapolation of aged data to populations

Because only a small proportion of the sampled population was aged, an ALK was used to transfer the variability in the radiocarbon aged samples across to the entire sampled population [35]. Age-length keys have been used for providing age estimates for a larger sample of fish based on a sub–sample of aged individuals [36]. Although using single morphological measurements (i.e. fish length) are not ideal for fish age estimations, additional co–variant measurements on fish scale were not feasible as the scales vary in size within and between fish specimens [37].

The ALK was developed from the sub–sampled aged population from each river. A normal probability distribution based on the 95% confidence limits was applied to each aged fish [38–39]. The normal distribution of probable ages for each aged fish was constructed by using the NORM.DIST function in Microsoft Excel, with probabilities for age classes around the assigned age equaling a total of one. Where two or more age probability distributions overlapped or where there were more than one fish per 50 mm fish length bin, the probabilities for each age class in that bin with a probability >0 were summed and divided by the total such that the overall probability within that 50 mm length bin still totaled to a value of one. The probability distribution for each fish length bin in the ALK for each river was therefore based on the aged individuals, the width of the normal distribution about those ages and the span between the aged individuals [39]. This produced a probability distribution for each 50 mm fish length bin that was applied to all non–aged fish. To determine if the resultant age distributions for each river derived from the ALK was different, the age frequency data were analyzed with a 2–sample Kolmogorov-Smirnov test [32].

To test the robustness of the ALK, age frequency distributions of all aged samples of fish <850 mm from the Burnett River (n = 43) were compared with the estimated age frequency of the same fish <850 mm generated using the ALK based on only 8 fish from the original stratified sampling of each 50 mm fish length bin. Differences in frequency distributions were analyzed with a 2–sample Kolmogorov-Smirnov test [32].

## Results

### Validation of aging method–Accounting for food impacts

**Fig 6:**
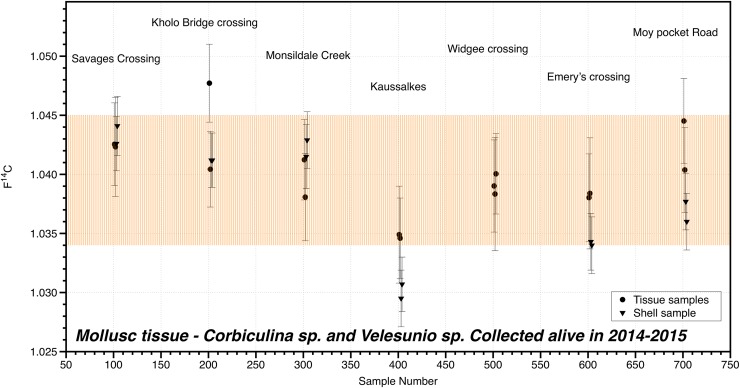
F^14^C of lungfish food. F^14^C values for mussel tissue and shell material from 7 locations. The highlighted box shows the range (2012–2015, due to mussels living for several years) in the atmospheric F^14^C CO_2_ for comparison.

Freshwater mussel tissue samples contained F^14^C values ranging between 1.035–1.047 (Fig 6), while the mussel shell carbonate F^14^C values ranged between 1.034–1.044 (Fig 6). The corresponding atmospheric CO_2_ F^14^C ranges from ~1.036–1.044 F^14^C between 2012–2015 are highlighted (Fig 6, [40]). These results demonstrate that a major food source for lungfish, freshwater mussels, does not contain older, more depleted sources of carbon that would potentially be metabolized by lungfish and affect the resultant age estimations from radiocarbon dating.

### Validation of Method–OTC tagged fish

**Fig 7:**
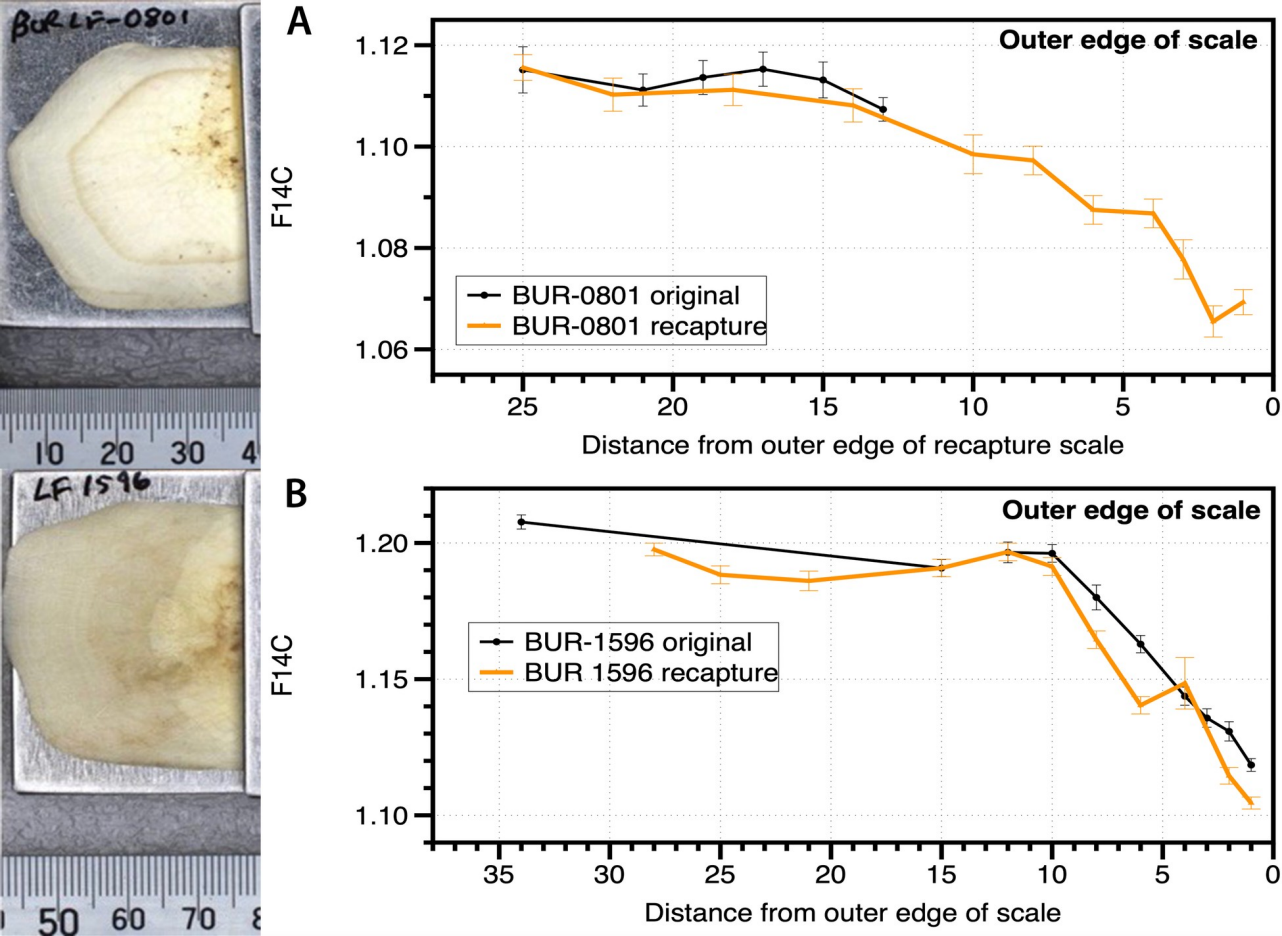
Oxytetracycline stained fish scales and F^14^C. A) Original fish 745 mm long, on recapture fish 1010 mm long, OTC mark visible on scale. B) Original fish 1036 mm long, on recapture fish 1060mm long, OTC mark not visible on scale.

Examination of the scales from the four OTC–marked lungfish showed differences in the appearance of the OTC mark (Fig 7). Under both visible and ultraviolet light, the OTC mark was clearly visible in the two fish injected as smaller individuals (LF0801, LF0910) (Fig 7A). In contrast, the OTC mark was not visible on any scales from the two larger individuals tagged (Fig 7B). The results from multiple measurements of F^14^C along the fish scale, from both a small and large fish at first capture, were plotted against distance from the edge of the scale. The data was referenced to the recaptured scale as this was significantly larger for the smaller fish at first capture (Fig 7). For the smallest fish at first capture (LF0801), the F^14^C results are plotted such that the original capture data overlays where the OTC mark on the recaptured scale was measured (~14 mm from the scale edge). The recapture data for this fish continues to show decreases in F^14^C consistent with the decrease in atmospheric CO_2_ F^14^C until recapture and both the capture and recapture scales show near identical F^14^C patterns. The resultant date of birth (1992) and date of tagging (1999) for this fish (LF0801) obtained through use of the radiocarbon reference curve were consistent and within measurement error.

The larger lungfish at first capture (BUR1596) only grew 24 mm in length in nearly 13 years. No OTC mark is visible on the recaptured scale, indicating the scale did not extend over this time period, which is consistent with little to no somatic growth in the fish (Fig 7B). The F^14^C results show near identical estimates of fish birth (1975) from both scales. Along with the lack of OTC mark on these scales, there are higher F^14^C values on the edge of the scales when compared to the atmospheric F^14^C values at time of recapture (Fig 7B). This suggests that this fish may have stopped growing at or near the time of tagging, with the difference in fish growth potentially due to measurement error of fish length [13].

These results show that F^14^C values on the edge of the scales are in close approximation to F^14^C value of atmospheric CO_2_ when the fish is still actively growing. Fish larger than ~1000 mm (17–53% of population dependent on river system (Fig 7) appear to grow very slowly, if at all. As the scale of these larger fish does not grow, the outer edge of the scale has a F^14^C value of the atmospheric CO_2_ at the time when growth ceased.

### Fish length frequency distributions

**Fig 8:**
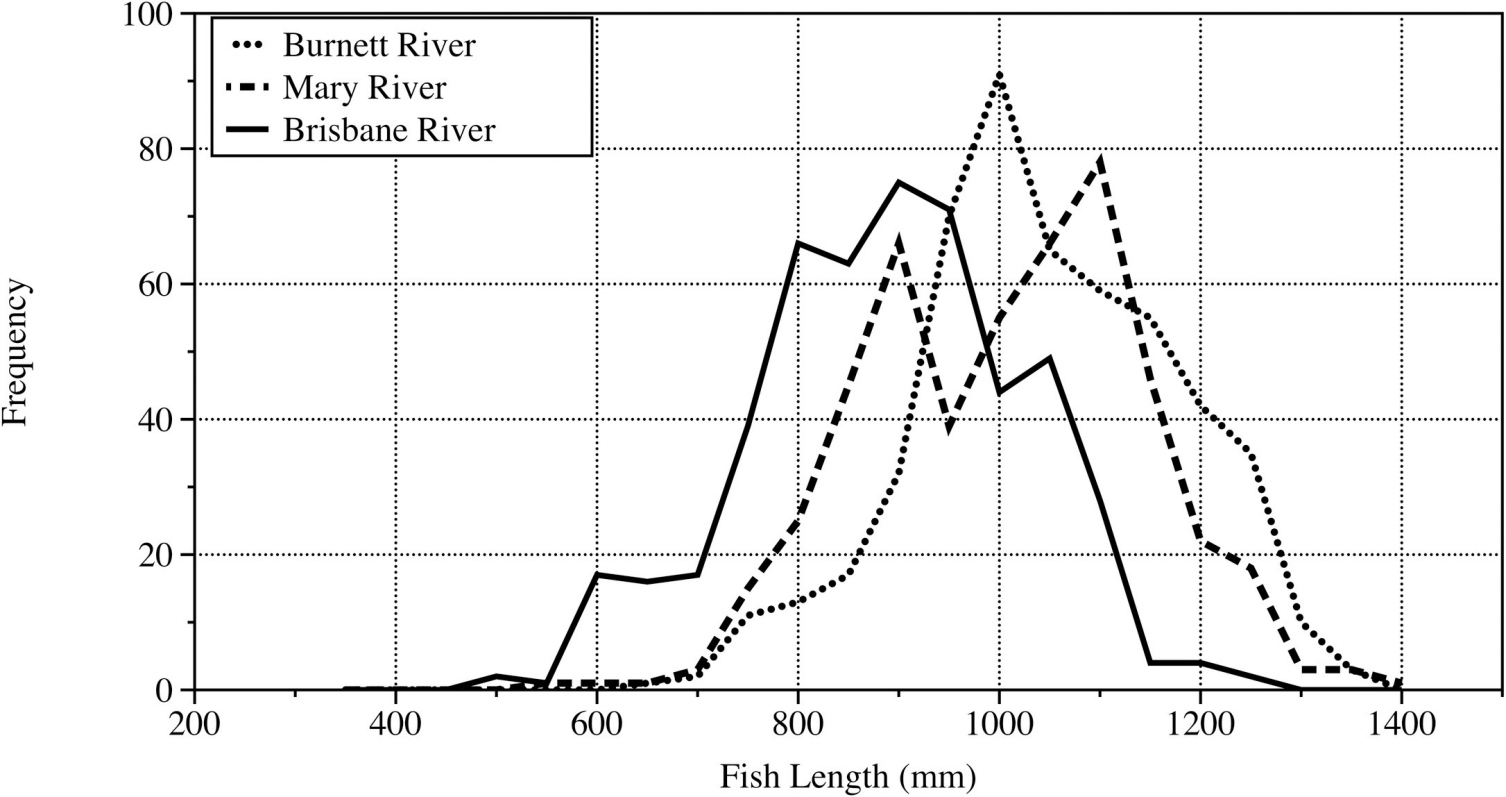
Length frequency histogram of Australian lungfish sampled from each river.

The length frequency distribution of the fish sampled from the 3 rivers is shown in Fig 8, highlighting the different length distributions for each river. The Burnett River distribution is skewed toward larger fish with a length peak around 1100 mm (Fig 8). The Mary River population is dominated by two modes at 900 mm and 1100 mm, with relatively more fish of a larger size (Fig 8). In contrast, the Brisbane River population has a relatively uniform distribution centered around 900 mm (Fig 8). Both the Mary River and Burnett River populations contain few fish below 750 mm in length (Fig 8). The two–sample Kolmogorov-Smirnov test confirmed that differences between length frequency distributions of all rivers were significant (p<0.0001).

### Age estimates

**Fig 9:**
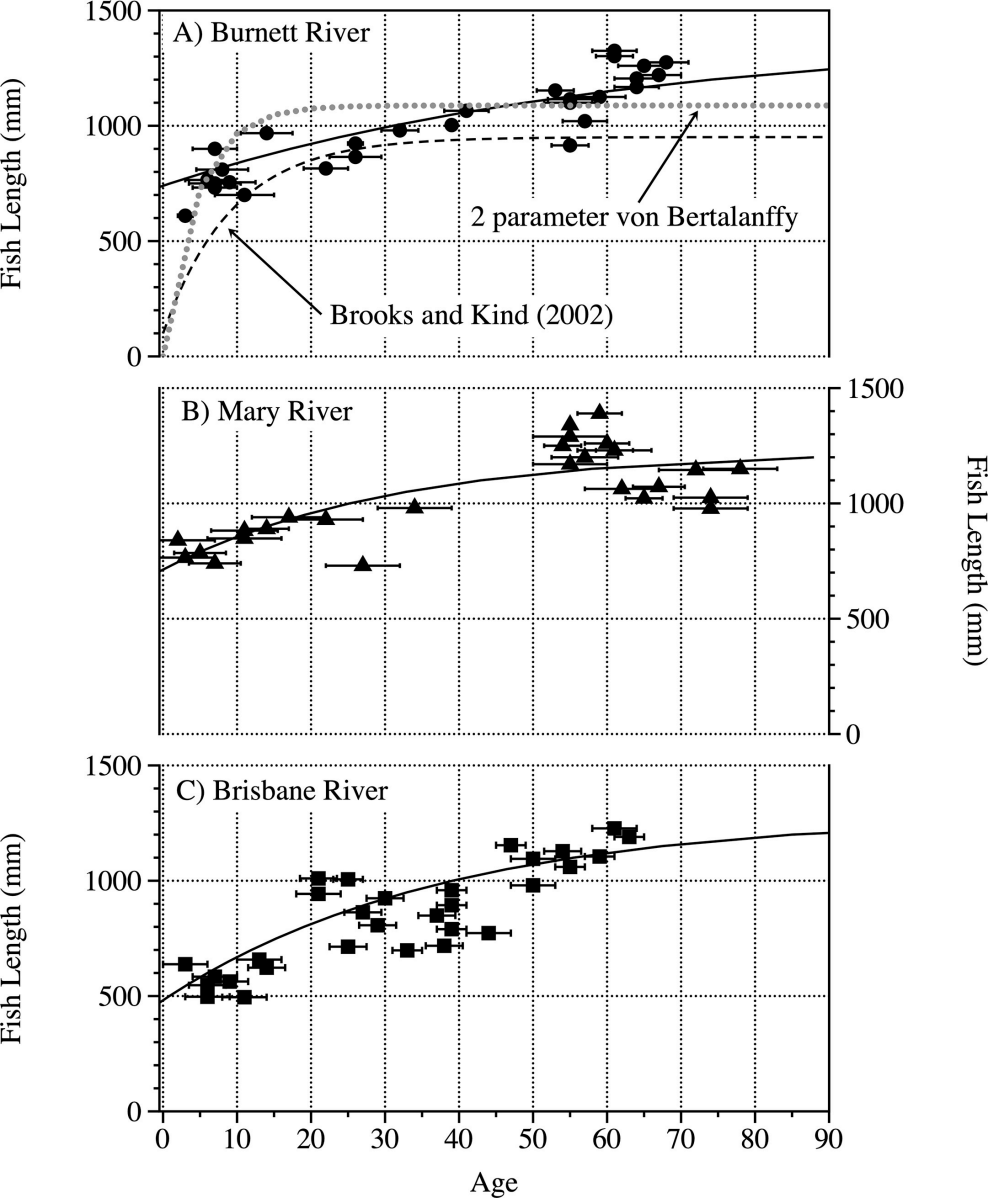
Size at age data for Australian lungfish. The results from the Burnett River (a), Brisbane River (b) and Mary River (c). The 3 parameter VBGF curves (solid lines) for each river are displayed. For the Burnett River, a previous 2 parameter VBGF mark–recapture curve [13] (dashed line) and a 2 parameter VBGF curve (dotted line) based on the current data are plotted for comparison.

The birth year probability distributions (from OxCal) are shown for each river in Fig 5 and S2 Table. In order to determine whether the birth year was before or after the “bomb” peak we measured multiple samples from each scale. Using the lungfish radiocarbon reference curve coupled with OxCal resulted in the 85 lungfish having ages that ranged from 2.5 to 77 years (Figs 5 and 9, S2 Table). Whilst the minimum age for each river was similar (2.5–3.5 years), the maximum ages varied more significantly, from 58 years in the Brisbane River to 77 years in the Mary River (Fig 9, S2 Table). The 95% confidence limits for these age estimate also varied significantly from 0.5 years for very young individuals to 5 years for older fish (Fig 9, S1 Table and S2 Table contain all the age data) [22, 31].

Analysis of the 3 parameter VBGF models for the three rivers revealed compared with the pooled data revealed that there are significant differences in parameters between rivers (F = 14.6, p<0.001) (Table 2). The lower AIC_c_ values found in each comparison highlighted that the models for the individual rivers fitted the data more appropriately with ΔAIC_c_ values >6 in all cases highlighting that the pooled model had little or no support **[33]**. Due to low samples sizes and no sex differentiation within the datasets, there is significant dispersion in the data with the potential for these growth parameters to be updated should additional aging data become available **[33]**.

**Table 2:** von Bertalanffy growth function parameter estimates for Australian lungfish.

River	Parameter estimate			AIC_c_	
	*L*_*∞*_ (mm)	*k* (/yr)	*t*_*0*_ (yr)	Individual model	Pooled model
Brisbane	1234	0.027	-17.1	**291.54**	302.87
Mary	1281	0.032	-27.1	**259.92**	265.57
Burnett	1400	0.016	-47.2	**246.21**	252.59

The 2 parameter VBGF curve was plotted on the length at age data for the Burnett River to compare with the 3 parameter VBGF curve for the Burnett River (Table 2) and another 2 parameter curve derived previously based on mark–recapture data (Fig 9) [13]. Whilst visual comparisons between tagging data and length at age data VBGF curves have been made in the past [41], any comparisons should be treated with caution as the data used to derive the models is different [42]. The asymptotic length derived by the 2 parameter VBGF for the Burnett River for the length at age data (*L*_*∞*_ = 1088 mm) was similar to that of the 2 parameter VBGF curve from mark–recapture (*L*_*∞*_ = 951 mm) (Fig 9) [13]. The growth coefficient (*k*) for the 2 parameter VBGF for the Burnett River for the length at age data (0.22) was also similar to that of the 2 parameter VBGF curve from mark–recapture (0.11) [13]. Both of the 2 parameter VBGF curves, however, produce *L*_*∞*_ values substantially less than the maximum sizes of fish sampled both in this study (Burnett = 1330 mm, Mary = 1390 mm, Brisbane = 1220 mm) (Fig 8) and that of the previous study that conducted the mark–recapture (1420 mm) [13]. In contrast, the 3 parameter VBGF curves fitted the length at age data more appropriately across the span of data, had equal residuals about the curves and produced *L*_*∞*_ values more closely reflective of the maximum size of fish captured (Table 2, Fig 8). A test of the most parsimonious model for the Burnett River highlighted that the 3 parameter VBGF model had the lowest AIC_c_ value (246.21) compared to the 2 parameter VBGF model (283.0). The AIC difference (ΔAIC) was >10, suggesting that the 2 parameter VBGF model had little or no support [33].

### Age distribution for sub–sampled fish

**Fig 10:**
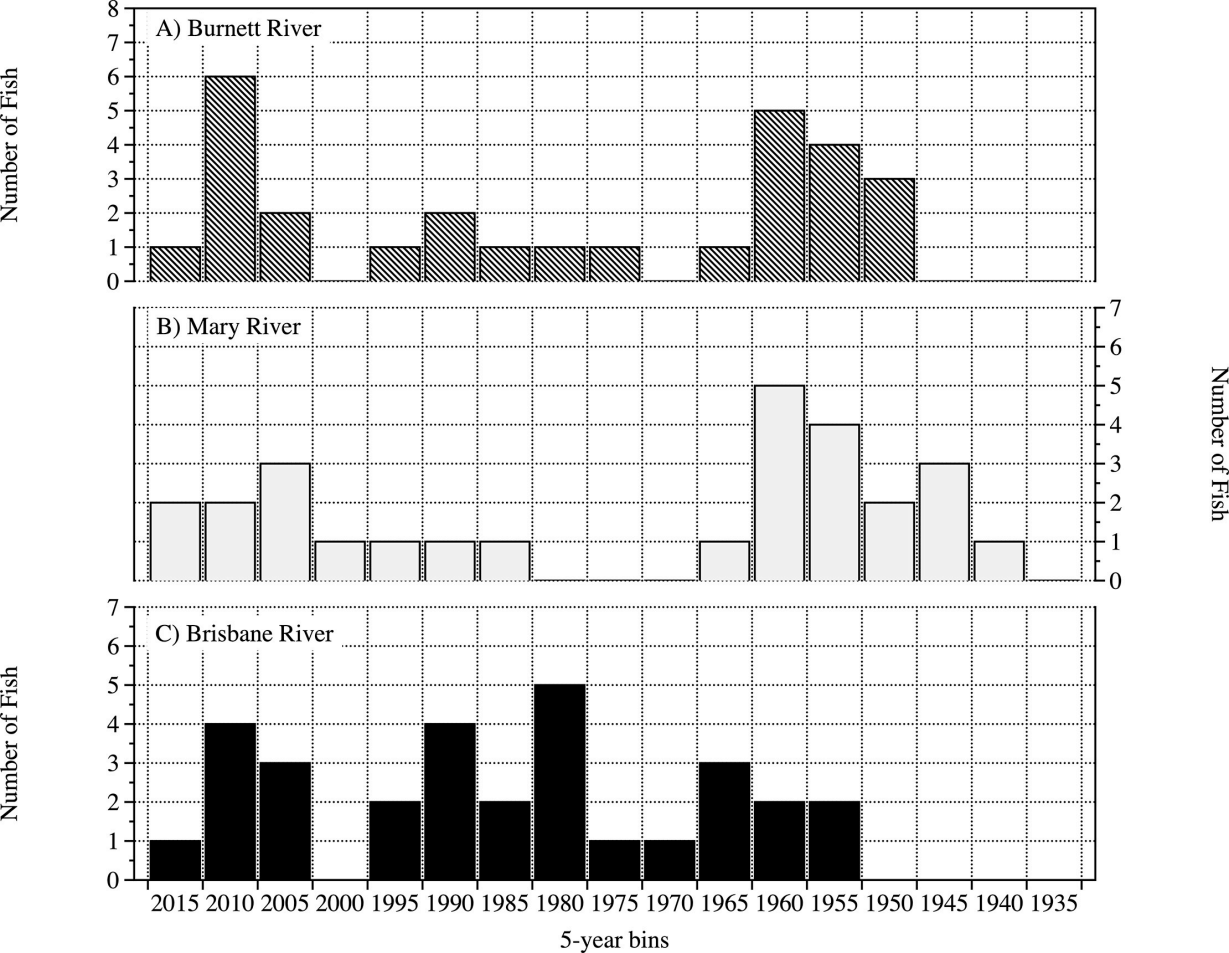
Age histogram of sub–sampled Australian lungfish. Results are shown from the Burnett River (a) (n = 28), Mary River (b) (n = 27) and Brisbane River (c) (n = 30). The 5 year bins end in the year labelled on the x axis.

The age distribution for the three rivers from the sub–sampled fish highlights three different patterns visible in the data (Fig 10). In the Burnett River, there is a generally consistent pattern of age structure for the last 60 years with the exception of two periods (1996–2010 and 1950–1960) (Fig 10A). The Mary River shows an even more distinct bi-modal distribution with a large gap in ages of fish between 1966 and 1980 (Fig 10B). There is a more even distribution of fish in each of the age classes in the Brisbane River except for one 5–year period up to the year 2000 (Fig 10C).

Even though visual differences in the age distribution data are apparent, because of the small sample sizes used for aging lungfish from each river, these differences were not globally statistically significant. The two–sample Kolmogorov-Smirnov test revealed that there is no significant difference in age distribution between the Burnett and Brisbane rivers (D = 0.297, p = 0.125) or between the Burnett and Mary rivers (D = 0.164, p = 0.818), yet there is between the Brisbane and Mary rivers (D = 0.425, p = 0.007). The Kruskal–Wallis test revealed that globally there is not significant differences between river systems (p = 0.147). Pairwise Mann–Whitney tests between river systems also revealed no significant differences in ages between Burnett and Brisbane rivers (p = 0.300), Burnett and Mary rivers (p = 0.316) but near significant differences between the Brisbane and Mary rivers (p = 0.059).

### Age data extrapolated to each population

**Fig 11:**
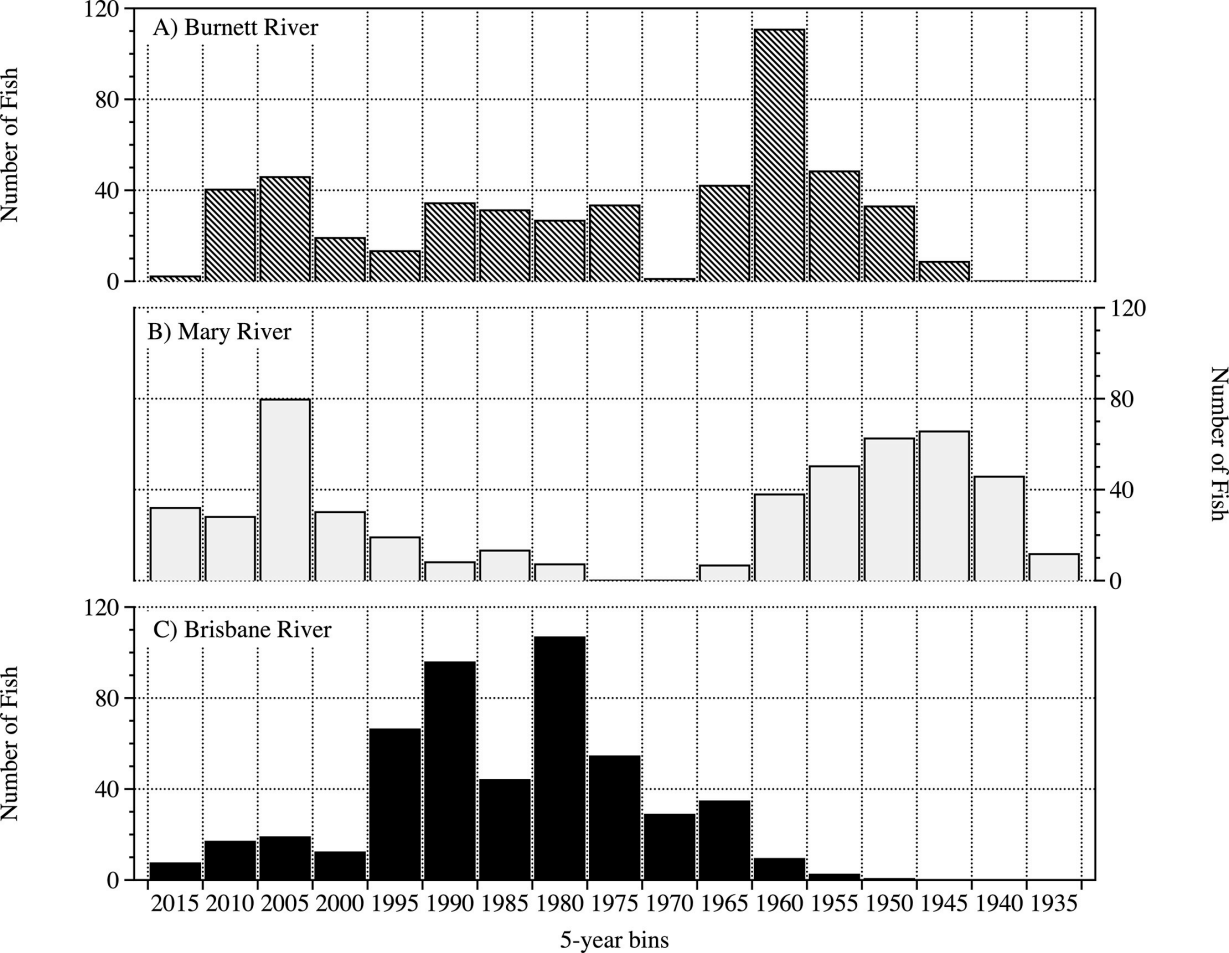
Histogram of estimated ages for the Australian lungfish populations. Results are shown from the Burnett River (a), Mary River (b) and Brisbane River (c). The 5 year bins end in the year labelled on the x axis.

Application of the ALK to the overall sample of 500 fish revealed the effect of the more abundant fish sizes classes in each population (Fig 11). There was a relatively more consistent age distribution in the Burnett River, with the 1955–1960 period being more dominant (Fig 11A). The bi-modal length frequency of the Mary River population and more variable age structure translated to a population age structure with a significant paucity of fish ages between 1965 and 1980 (Fig 11B). The greater abundance of fish in the Brisbane River in the 600–900 mm size range and relatively consistent age distribution in the sub–sampled population produced a population age structure that is clearly modal towards middle age classes and more reflective of the normal distribution of the length data (Figs 8 and 11C). The two–sample Kolmogorov-Smirnov test revealed significant difference in age distribution between all rivers when applied to the population of 500 fish: Burnett and Brisbane rivers (D = 0.454, p<0.001), Burnett and Mary rivers (D = 0.303, p<0.001) and Brisbane and Mary rivers (D = 0.541, p<0.001).

As the population age data is calculated from a combination of aged individuals with known aging error, and is applied across the individual populations via an ALK, there is high probability of error in the final age distribution due to low sample sizes. However this technique of using ALKs is well utilized in fisheries science where there are numerous samples of fish lengths but limited aging information due to limitations (cost and time to process samples) [38].

**Fig 12:**
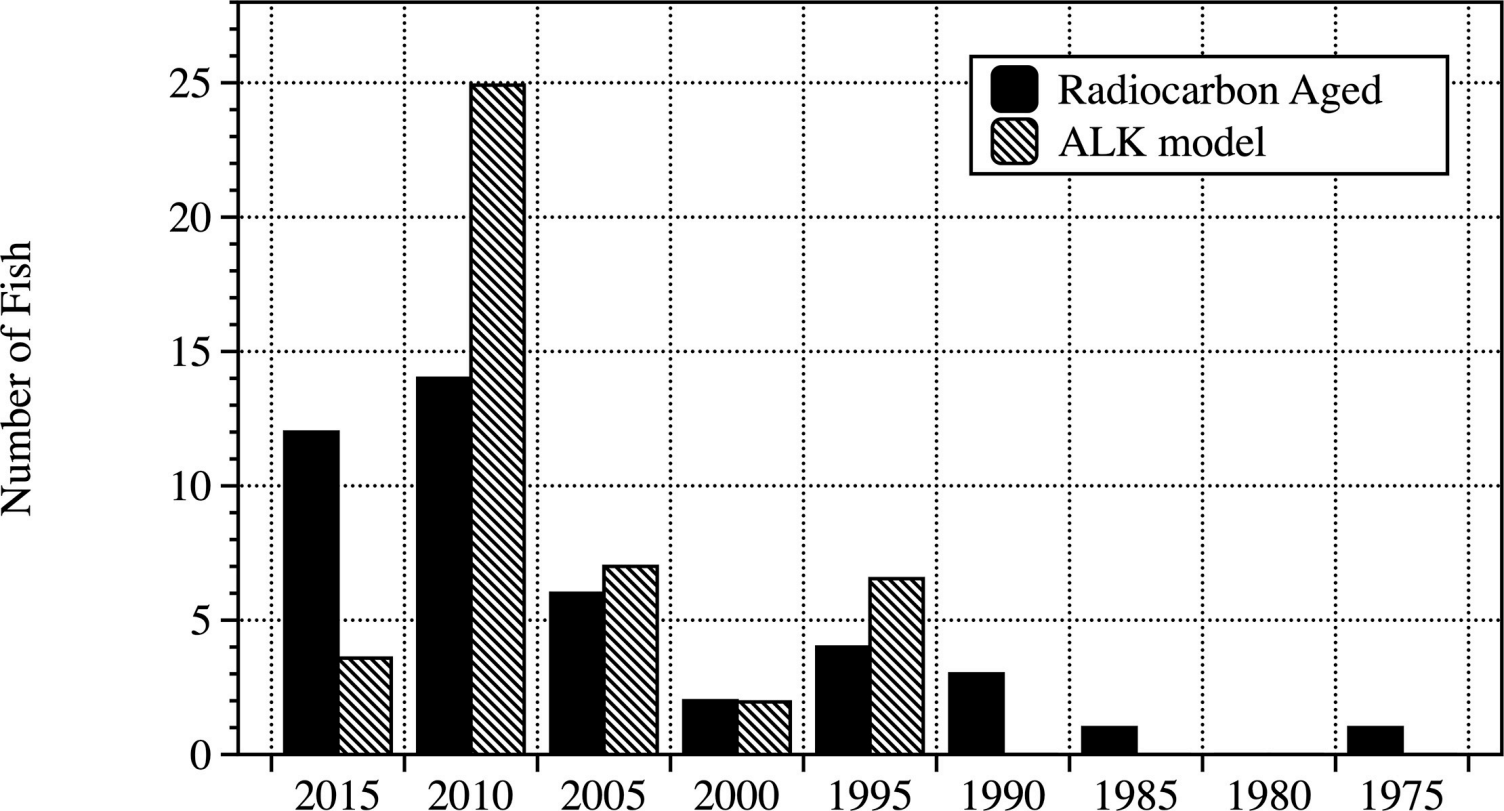
Comparison of the actual age frequency of lungfish with those derived from the ALK. Results from fish <850 mm in length from the Burnett River derived from radiocarbon aging and that derived using the ALK of the sub–sampled population.

All lungfish <850 mm from the Burnett River were radiocarbon aged to further refine the age structure of younger fish in an effort to resolve recent lungfish recruitment variability (n = 43). When compared to the estimated age frequency of all fish <850 mm from the Burnett River from ages calculated from the ALK, based on the eight aged individuals in the sub–sampling, no significant differences were found between the groups (D = 0.199, p = 0.311) (Fig 12). This shows that using the ALK to estimate the population structure is an appropriate strategy and is unbiased, at least in fish <850 mm.

## Discussion

We have shown that aging of the threatened Australian lungfish can be successfully completed using bomb radiocarbon dating of scales, with an entire modern curve visible from scales taken from older lungfish [14]. Using this technique, we were able to age lungfish from 2.5 to 77 years, confirming untested postulations of longevity and importantly, evidence of recent and continual recruitment in all three rivers totaling its current distribution.

### Validation

As this is a novel technique, validation of the aging approach through multiple lines of evidence is important [43]. One issue with using bomb radiocarbon dating is that the basal food sources can affect the resultant radiocarbon level in the consumer/predator [20]. The results of the dating of freshwater mussel shell and muscle material indicate that this lungfish food source is composed of, and is itself feeding on, modern carbon. The carbon isotope fraction of collagen, chitin and insoluble organic fractions of mussel shells are related to the isotopic composition of the diet [44]. Although there is a possibility that the major food source for lungfish and the resultant ages from radiocarbon dating may be affected by depleted carbon sources [20], results from the current study show this was not the case. Aging studies conducted on deep–water marine species have documented depleted radiocarbon from stratified deeper oceanic waters, and consequential trophic accumulation, causing a lag in the peak of the radiocarbon curve or an overall reduction in radiocarbon values [20, 45]. The results from the mussel aging have confirmed that there is no evidence of the “freshwater reservoir effect”, or older depleted sources of carbon [46].

Reference curves using known time periods of key inflections of the atmospheric radiocarbon curve have been incorporated in numerous aging studies [22, 31]. For lungfish, there were no samples of known–age fish from known time periods that could be used to assist in constructing reference curves [17–18]. Instead, a number of OTC-marked individuals recaptured after 10–13 years provided a basis for further validation. The OTC marking highlighted that the scale F^14^C values matched those of atmospheric radiocarbon at the time of marking, and on the edge of the scale, in the smaller tagged individuals upon recapture. This highlights that the radiocarbon source for lungfish matches more closely with atmospheric sources as documented for other freshwater fish species [18]. Furthermore, the lack of OTC mark in the larger individuals suggests reduced metabolism in the organism post–tagging and is confirmed by limited growth (within measurement error) [13]. Higher F^14^C values found on the edge of these scales compared to ambient levels at time of sampling further emphasizes cessation of growth [13]. Cessation of growth in fish has been identified in long–lived marine species such as porbeagle sharks (*Lamna nasus*) [47] and White sharks (*Carcharodon carcharias*) [48]. Our study has shown that the scales of lungfish cease to grow during their lifetime and do not continue to grow as previously thought [27].

As the peak F^14^C levels seen in lungfish scales are a fraction of the atmospheric F^14^C, there was a need to construct a reference radiocarbon curve. As opposed to coral reef fish aging studies where there is a wealth of radiocarbon chronologies available for coral reefs to use as a reference curve [49–50], there is no global freshwater fish equivalent. The radiation chronology for freshwater drum (*Aplodinotus grunniens*) has been used as a reference for aging of pallid sturgeon (*Scaphirhynchus albus*) [16]. Bomb radiocarbon dating has been conducted on other freshwater fish species in the past based on otoliths and fin spines [16–18, 19, 51], however this is the first species of fish either in freshwater or marine environments aged using fish scales. For another freshwater fish species, the ^14^C reference chronology for freshwater Arctic species was found to more closely resemble the atmospheric radiocarbon curve rather than one derived from otoliths from marine finfish [18]. There is suggestion that this strong influence of atmospheric input of radiocarbon into freshwater systems even extends to estuarine habitats [19].

### Differences in lungfish growth between rivers

Differences in the length frequency and age structures of lungfish between rivers is thought to be a combination of a number of factors. All three populations are genetically separate from each other and are currently affected by different levels of water regulation [7, 10]. The Mary River has minimal impediments along its watercourse whereas the Burnett River has multiple water storages in its catchment. The streamflow in the Mary River is naturally highly variable, whereas the flows in the Burnett River are now more regulated and may reduce the impacts of smaller floods on the species [10]. The Brisbane River sample reach has a large dam upstream of the sampling sites that provides regular flow downstream [10]. The abundance of smaller/younger fish and more normal distribution of fish length and ages of fish in the Brisbane River may be a reflection of consistent recent recruitment facilitated by more constant stream flows since the construction of Wivenhoe Dam (1982) [52]. In contrast, river damming has been blamed for a lack of recruitment for a similarly threatened riverine freshwater fish species, the pallid sturgeon from the Missouri River (USA) [16]. As few adult sturgeon exist, radiocarbon dating has been used on this freshwater fish to validate other aging techniques and to establish whether the remnant population was the result of spawning events prior to closure of the last dam [16].

Derived growth models for lungfish highlighted significant differences, with the Brisbane River demonstrating a lower asymptotic length than that of the Burnett and Mary rivers. The Brisbane River population was founded from a small number of individuals translocated from the Mary River ~100 years ago, therefore the smaller *L*_*∞*_ estimate for this river may be a genetic artefact of low intraspecific genetic variation from founding individuals or due to absence of older fish, as suggested in this current study [7, 53]. This reach of the Brisbane River also has a relatively high abundance of lungfish, suggesting potential density-dependent factors affecting growth in this sub–population [54]. Low sample sizes of radiocarbon aged individuals mean that the growth models estimates are reflective of the dispersion of the age data and the estimates could be improved by using techniques for other threatened species such as back–calculation [33].

The relatively flat growth curves produced from the age data produced in this study reflect the paucity of juveniles included in VBGF curve development. Similar, near–linear VBGF curves, with very negative *t*_*0*_ values (-40.29) and low *k* values (0.0289) have been documented for other initially fast growing, long–lived fish species where these is a paucity of young fish aged [41]. In addition, a recent comparison between 2 and 3 parameter VBGF models highlighted that even though the 2 parameter model was thought to work better in data–sparse studies, even slight deviations in the fixed length at age zero (*L*_*0*_) can cause considerable bias in the estimated growth parameters [55]. The comparison showed that the use of these biased growth parameters could have profound consequences for resultant fisheries stock status assessments [55]. Although the 2 parameter VBGF curve can have utility where there is a lack of juveniles in the age length data or the data is sparse, the resultant growth parameter *k* values may be less biased but will not reduce the uncertainty of the model [55]. The 3 parameter VBGF model was the chosen model in this study as it was a better reflection of the observed length at age data, was the most parsimonious model and produced the most biologically relevant *L*_*∞*_ values. However, the 2 parameter VBGF curve based on the age data more closely reflected a previous mark–recapture 2 parameter VBGF curve, may have a less biased estimate of the *k* parameter and reflect early growth, but produces a more biased *L*_*∞*_ value that is not biologically realistic. Extremely rapid growth in the first few years of life followed by slow growth may not be suitably represented by the von Bertalanffy growth curve and may be better represented by other growth models such as power or broken stick models [56]. As the overall aim of fitting a growth curve in this study was to describe the relatively flat observed length at age data (fish aged 2.5–77 years), there was no need to fit other types of models.

There are no external morphological differences evident between sexes in Australian lungfish, with sex only able to be determined by internal examination or by the presence of extruded ripe eggs [13]. Larger lungfish in the Burnett River (>1200 mm) are dominated by females (80%), with the average size at maturity being 834.4 mm and fully mature by 1000 mm [13]. Male fish are on average mature at a smaller size of 767.2 mm and also fully mature by 1000 mm [13]. As such, the large variation in length-at-age could be attributed to different growth rates between sexes as demonstrated in many other fish species [50, 56]. Until genetic techniques are available to differentiate sex, or less intrusive field techniques are available, broad scale sexing of lungfish will not be possible to further age differences between sexes.

The lack of small lungfish captured during this study has been an ongoing issue since their discovery in the 19^th^ century when absence of fish less than 2.2 kg (approximately 630 mm) was first documented [57]. Without being able to age this species in the past, there has been no way to understand recruitment patterns. Our data suggests that there has been recruitment in all three rivers within the last decade to varying degrees. Whilst there has been spawning observed over this time [9, 58], there has also been concern over the poor development and abnormality of embryos, hatchlings and juveniles [59]. Further investigation is being undertaken to understand the shorter and longer term recruitment drivers for the Australian lungfish. By now having the ability to age the species, there is the possibility to review the success of previous recruitment events by correlating hydrological and other environmental drivers over time.

## Supporting information

S1 Table Contains the measured radiocarbon values for each individual sample from each scale. (CSV)

S2 Table Contains the fish collection information and age data for each fish analyzed in this study. (XLSX)
